# Polymorphisms in gene *MTHFR* modify the association between gestational weight gain and adverse birth outcomes

**DOI:** 10.3389/fnut.2022.919651

**Published:** 2022-08-08

**Authors:** Weixiang Wu, Dan Luo, Xiaolin Ruan, Chunming Gu, Weiming Lu, Kailing Lian, Xiaoping Mu

**Affiliations:** ^1^Department of Clinical Laboratory, Guangdong Women and Children Hospital, Guangzhou, China; ^2^Department of Preventive Medicine, School of Public Health, Guangzhou Medical University, Guangzhou, China; ^3^Medical Genetics Center, Guangdong Women and Children Hospital, Guangzhou, China

**Keywords:** gestational weight gain, methylenetetrahydrofolate reductase polymorphisms, low birth weight, macrosomia, small-for-gestational age, large-for-gestational age

## Abstract

Evidence suggests a potential relationship between gestational weight gain (GWG) and adverse birth outcomes. However, the role of maternal genetic polymorphisms remains unclear. This study was conducted to investigate whether the relationship of GWG with risk of adverse birth outcomes was modified by methylenetetrahydrofolate reductase (*MTHFR*) polymorphisms. A total of 2,967 Chinese pregnant women were included and divided into insufficient, sufficient, and excessive groups based on the Institute of Medicine (IOM) criteria. Polymorphisms of C677T and A1298C in gene *MTHFR* were genotyped. Multivariable logistic regression models were introduced after controlling major confounders. Excessive GWG was found to increase the odds ratio (OR) for macrosomia [OR = 3.47, 95% confidence interval (CI): 1.86–6.48] and large-for-gestational age (LGA, OR = 3.25, 95% CI: 2.23–4.74), and decreased the OR for small-for-gestational age (SGA, OR = 0.60, 95% CI: 0.45–0.79). Pregnant women with insufficient GWG had a higher frequency of SGA (OR = 1.68, 95% CI: 1.32–2.13) and a lower rate of LGA (OR = 0.51, 95% CI: 0.27–0.96). Interestingly, significant associations of GWG categories in relation to low birth weight (LBW), macrosomia, and SGA were only suggested among pregnant women with *MTHFR* A1298C AA genotype. Among pregnant women with insufficient GWG group, an increased risk of 3.96 (95% CI: 1.57–10.01) for LBW was observed among subjects with the A1298C AA genotype, compared to the AC+CC genotype group. GWG categories are closely related to LBW, macrosomia, SGA and LGA, and the associations were modified by the polymorphism of *MTHFR* A1298C.

## Introduction

Excessive weight and obesity have become major public health issues worldwide, especially for pregnant women who are in a special physiological condition. Nearly 31.8% of US women of reproductive ages are obese (1), and the prevalence of overweight and obesity for Chinese women aged 18–44 years are 26.4 and 11.0% (2). Gestational weight gain (GWG) is a valuable indicator of maternal nutritional status, reflecting maternal fat storage and maintaining the growth of the fetus, placenta, and uterus. In 2009, the Institute of Medicine (IOM) provided specific recommendations regarding the optimal GWG accounting for pre-pregnancy body mass index (BMI) categories (3). According to the data from the US Center for Disease Control, 48% of pregnant women gained excessive GWG, and 21% gained insufficient GWG (4). In China, excessive weight gain occurred in 57.9% of pregnant women, and insufficient weight gain was 12.5% (5). GWG is closely related to fetal growth restriction, pregnancy complications, fertility, pregnancy loss, infant mortality, and even childhood obesity.

Accumulated evidence suggests that genetic susceptibility is an important factor in fat accumulation and lipid metabolisms, possibly resulting in abnormal GWG for pregnant women. C677T and A1298C variants in the methylenetetrahydrofolate reductase (*MTHFR*) gene known to reduce enzyme function and ultimately lead to enhanced homocysteine levels were considered as potential candidates (6). It has been reported that irregular MTHFR activity can affect body fat storage *via* epigenetic mechanisms, as homocysteine plays key roles in the transfer of methyl groups in the activated methyl cycle (7). Several studies have investigated the potential associations of *MTHFR* C677T and A1298C polymorphisms with obesity/overweight or other metabolic syndromes (8–10). For instance, a Chinese study has reported significant weight gain related to C677T variants (11). A dietary study observed that *MTHFR* variants are associated with BMI at baseline, and obese individuals with C677T CC genotype lost more weight than the T allele carriers after nutritional intervention (12). Some researchers speculated that elevated homocysteine levels induced by *MTHFR* polymorphisms might affect the development of overweight/obesity through epigenetic control of gene expression in fat storage in the body, since methyl and homocysteine metabolism are closely related to DNA methylation (13, 14).

There is well-documented evidence on the associations between GWG and obstetric outcomes in human studies (9). However, the role of maternal genetic polymorphisms in these relationships requires further investigation. The present study aimed to investigate whether the relationship between GWG and adverse birth outcomes was modified by *MTHFR* C677T and A1298C polymorphisms, two loci in gene *MTHFR* that have been most widely investigated.

## Materials and methods

### Study population

This study was conducted at Guangdong Women and Children Hospital, a large teaching tertiary public hospital in Guangzhou city, Guangdong province, China. Pregnant women who delivered at the study hospital between January 2017 and December 2019 were considered for inclusion, and a total of 4,640 pregnant women were selected according to the following inclusion criteria: (i) > 18 years old, (ii) had regular prenatal examinations, (iii) gave a birth to singleton live baby, and (iv) had complete data on basic information, gestational weight during pregnancy, the genotype of *MTHFR* genes, and birth outcomes. In addition, we excluded women who underwent *in vitro* fertilization (*n* = 636), had multiple pregnancies (*n* = 340), stillbirths (*n* = 446), or abortions (*n* = 251). Finally, a total of 2,967 mother-infant pairs were included in the present study. This study protocol was approved by the Medical Ethical Committees of Guangdong Women and Children Hospital. All health care procedures were carried out in accordance with approved guidelines and regulations.

### Weight measurements

Maternal pre-pregnancy BMI was calculated as self-reported pre-pregnancy weight in kilograms divided by the square of height in meters (kg/m^2^). According to Chinese BMI classification, pre-pregnancy BMI were assigned as underweight (<18.5 kg/m^2^), normal weight (18.5–23.9 kg/m^2^), overweight (24.0–27.9 kg/m^2^), and obesity (≥28.0 kg/m^2^), respectively (15). GWG was defined as the difference between the pre-pregnancy weight and weight at delivery for pregnant women. The IOM recommendations were used to classify the GWG group, which were 12.5–18 kg for pregnant women that were underweight, 11.5–16 kg for normal weight women, 7–11.5 kg for overweight women, and 5–9 kg for obese women, respectively (3). Based on IOM criteria, women were divided into an insufficient group (GWG < the recommended range), a sufficient group (GWG within the recommended range), and an excessive group (GWG > the recommended range).

### Birth outcome and covariates

Anthropometric information of the newborns was extracted from the medical records. Once a newborn was born, obstetric nurses immediately determined and recorded its birth weight and length. Obstetric nurses abstracted gestational age, mode of delivery, infant sex, birth weight, length, and head circumference. In the present study, low birth weight (LBW) was defined as birth weight <2,500 g, and macrosomia was considered as birth weight >4,000 g. Based on the population-based birth weight reference percentiles for Chinese, birth weight was divided into small-for-gestational age (SGA; birth weight ≤ the gender-specific 10th percentile for gestational age) and large-for-gestational age (LGA; birth weight ≥ the gender-specific 90th percentile for gestational age) (16). Additionally, data on maternal age, gravidity, parity, education, smoking and drinking status, pregnancy complications [gestational diabetes mellitus (GDM) and hypertensive disorders in pregnancy (HDP)], and homocysteine were extracted for potential covariates.

### *MTHFR* genotype

Venous blood samples were collected using vacuum tubes with potassium salt of ethylenediaminetetraacetic acid and stored at 4°C. Genomic DNA was extracted from whole blood samples using a DNA extraction kit (Magen, Guangzhou, China) on an automated nucleic acid extraction workstation (Hamilton, Sweden), according to the manufacturer's instructions. *MTHFR* C677CT and A1298C variants were genotyped using fluorescence quantitative polymerase chain reaction (PCR), where each reaction system contained Premix Ex Taq (TakaRa, Japan), TaqMan-MGB probes, deionized water, and genomic DNA. For *MTHFR* C677CT, the forward primer sequence was 5′-CTCTTCTACCTGAAGAGCAAGTCC-3′, and the reverse primer sequence was 5′-CACTCCAGCATCACTCACTTTGT-3′. For *MTHFR* A1298C, the forward primer sequence was 5′-CCGAAGCAGGGAGCTTTG-3′, and the reverse primer sequence was 5′-CGGTGCATGCCTTCACAA-3′. Reaction conditions are 95°C for 3 min to activate fluorescent groups, following by 40 cycles of amplification (95°C for 20 s, 58°C for 20 s, 65°C for 45 s). The endpoint fluorescence was read and analyzed by a ViiA 7 Dx PCR system (Applied Biosystems, USA).

### Statistical analysis

First, general characteristics of our participants were described. Continuous variables were described as mean ± standard deviation or median (interquartile range). Categorical data were described by frequencies (%). The difference between the 3 GWG groups were compared using parametric or non-parametric methods for continuous or categorical data.

Multivariable logistic models were applied to assess the relationship between GWG categories and adverse birth outcomes, among which, pregnant women in sufficient group of GWG were considered the reference group. The cases included pregnant women who gave birth to LBW (*n* = 144), macrosomia (*n* = 49), SGA (*n* = 432), or LGA (*n* = 149) babies, and the controls were those who delivered infants without the above 4 birth outcomes (*n* = 2,365). We adjusted for potential covariates in the regression models based on previous reports or statistical considerations. The change-in-effect estimate method was applied to select confounders, in which covariates that changed the main effect estimates by ≥10% were introduced into the models. The inclusion of potential confounders in the final linear regression models for LBW and macrosomia was as follows: maternal age (continuous), education level (less than high school, high school or equivalent, and college or above), delivery mode (natural labor or cesarean section), parity (nulliparous or multiparous), gestational age at delivery (continuous), HDP (no or yes), GDM (no or yes), and infant sex (boys or girls). With regard to regression models for SGA and LGA, gestational age at delivery was not included in the case of co-linearity. To assess whether different pre-pregnancy BMI categories affect the observed associations, World Health Organization (WHO) criteria of BMI classification were introduced (BMI for underweight, <18.5; normal weight, 18.5–24.9 kg/m^2^; overweight, 25.0–29.9 kg/m^2^; and obesity, ≥30.0 kg/m^2^) and the analyses were repeated (17).

Deviation from Hardy–Weinberg expectation (HWE) was calculated among the control group, and *P*-value > 0.05 indicated that the two variants were in accordance with HWE. Co-dominant, dominant, recessive, and additive models were used to explore the associations between genetic polymorphisms and adverse birth outcomes. False discovery rate (FDR) corrections were introduced to adjust the *P*-values for multiple corrections. To investigate the potential modification effects of genetic polymorphisms on the associations between GWG categories and adverse birth outcomes, study subjects were divided into non-mutated and mutated groups, considering the dominating effect of mutated allele as well as for better statistical power, similar to previous studies (18, 19). The likelihood ratio test was used to analysis the interaction effect between GWG categories and genetic polymorphisms on birth outcomes. Differences in the likelihood scores of the two models with and without the interaction term of genotypes and GWG categories were compared. In addition, we further evaluated the associations of *MTHFR* polymorphisms and adverse birth outcomes within the strata of GWG categories. Generalized additive models were fitted to explore the dose-response relationship between GWG values and adverse birth outcomes with spline smoothing function among high-risk pregnant women with *MTHFR* A1298C AA genotype.

All analyses were performed using SAS version 9.4 (SAS Institute, Inc., Cary, NC, USA) and R version 3.3.3 (R Foundation for Statistical Computing). *P*-value < 0.05 (two-tailed) was considered statistically significant.

## Results

### Study population

**Figure 1:**
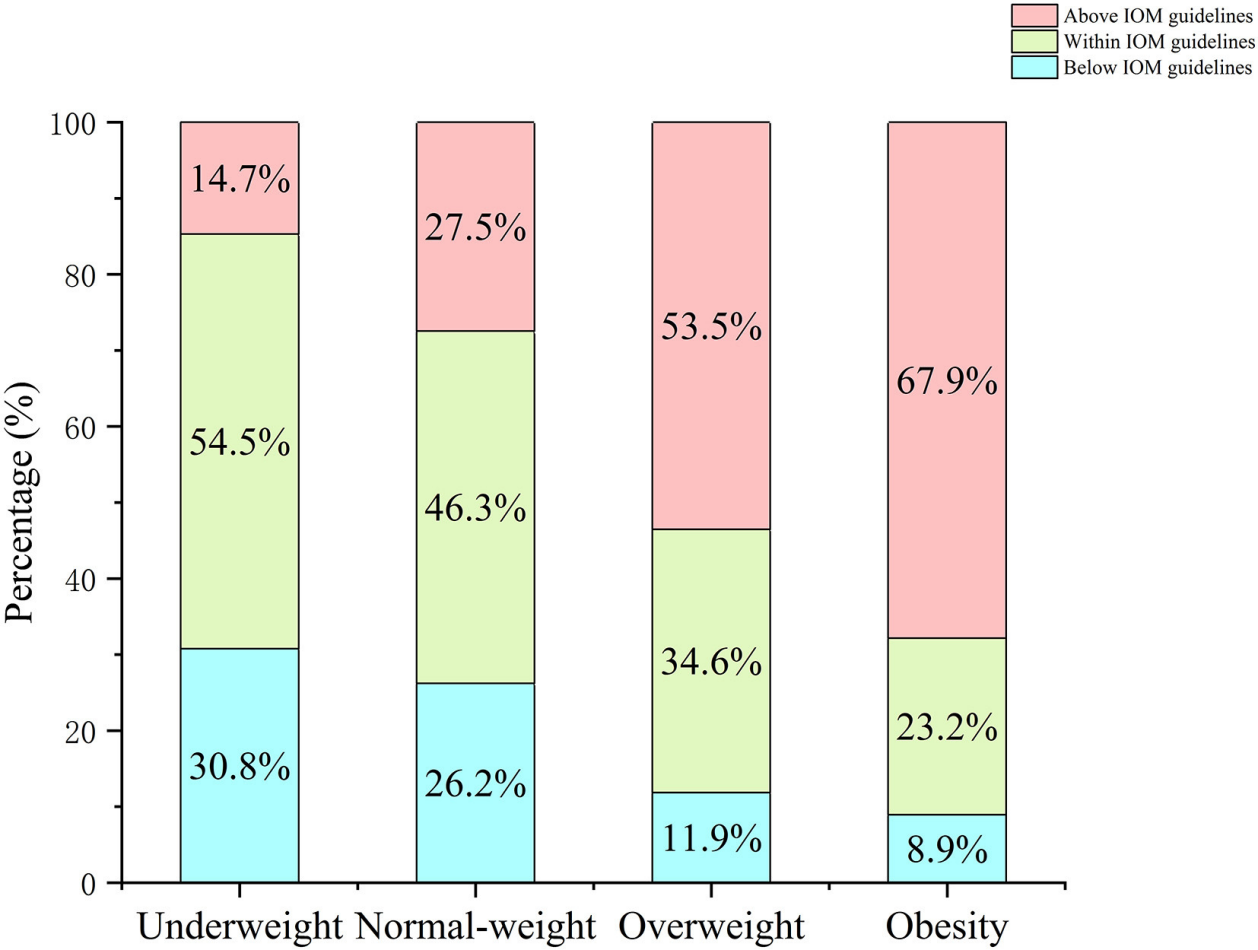
Percentage of pregnant women in each BMI category by IOM GWG guidelines. Pre-pregnancy BMI were categorized according to Chinese BMI classification (underweight, <18.5 kg/m^2^; normal weight, 18.5–23.9 kg/m^2^; overweight, 24.0–27.9 kg/m^2^; and obesity, ≥28.0 kg/m^2^).

Descriptive variables are listed in Table 1. A total of 2,967 mother-infant pairs were selected for analysis. The average of maternal age was 30.0 ± 3.9 years. For pre-pregnancy BMI, there were 2014 (67.9%) pregnant women in the normal-weight group, 585 (19.7%) in the underweight group, 312 (10.5%) in the overweight group, and 56 (1.9%) in the obesity group. At the time of delivery, the normal-weight group, underweight group, overweight group, and obesity group had 670 (22.6%), 2 (0.1%), 1,543 (52.0%), and 752 (25.3%) pregnant women, respectively. When grouped according to the IOM guidelines, there were 1,373 (46.3%) pregnant women with GWG within the recommended range, 750 (25.3%) below the recommended range, and 844 (28.4) above the recommended range. Underweight and normal-weight women were more likely to have GWG within the IOM guidelines, while overweight and obese women were more likely to have GWG above the IOM guidelines (Figure 1). Most mothers were nulliparous (*n* = 1,730, 58.3%), and the deliver mode of 1,082 (36.5%) women was cesarean section. The present study reported that none of the mothers had a history of smoking or drinking during pregnancy. Additionally, 132 (4.4%) and 461 (15.5%) mothers were diagnosed with HDP and GDM, respectively, in this population. There were 51.6% (*n* = 1,531) boys and 48.4% (*n* = 1,436) girls among the infants. The mean weight, length, and gestational age at birth were 3202.5 ± 427.9 g, 49.5 ± 1.9 cm, and 39.2 ± 1.4 weeks, respectively.

**Table 1:** Basic characteristics of study population according to the IOM guidelines (*n* = 2,967).

**Characteristics**	**Total** **(*n* = 2,967)**	**Below** **(*n* = 750)**	**Within** **(*n* = 1,373)**	**Above** **(*n* = 844)**	***P*** **-value**
Mothers					
Maternal age (years)	30.0 ± 3.9	30.4 ± 4.1	29.9 ± 3.8	29.9 ± 4.1	0.016
Pre-pregnancy BMI (kg/m^2^)	20.8 ± 2.8	20.5 ± 2.5	20.4 ± 2.6	21.8 ± 3.1	<0.001
Pre-pregnancy BMI (kg/m^2^)					
Underweight (<18.5)	585 (19.7)	180 (24.0)	319 (23.2)	86 (10.2)	<0.001
Normal-weight (18.5–23.9)	2,014 (67.9)	528 (70.4)	933 (68.0)	553 (65.5)	
Overweight (24.0–27.9)	312 (10.5)	37 (4.9)	108 (7.9)	167 (19.8)	
Obesity (≥28.0)	56 (1.9)	5 (0.7)	13 (0.9)	38 (4.5)	
Pregnancy BMI (kg/m^2^)	26.3 ± 3.1	24.1 ± 2.2	25.8 ± 2.2	29.0 ± 3.0	<0.001
Pregnancy BMI (kg/m^2^)					
Underweight (<18.5)	2 (0.1)	2 (0.3)	0 (0.0)	0 (0.0)	<0.001
Normal-weight (18.5–23.9)	670 (22.6)	372 (49.6)	288 (21.0)	10 (1.2)	
Overweight (24.0–27.9)	1,543 (52.0)	353 (47.1)	853 (62.1)	337 (39.9)	
Obesity (≥28.0)	752 (25.3)	23 (3.1)	232 (16.9)	497 (58.9)	
Education level					
< High school	149 (5.0)	32 (4.3)	74 (5.4)	43 (5.1)	0.763
High school	405 (13.7)	105 (14.0)	180 (13.1)	120 (14.2)	
≥College	2,413 (81.3)	613 (81.7)	1,119 (81.5)	681 (80.7)	
Parity					
Nulliparous	1,730 (58.3)	432 (57.6)	810 (59.0)	488 (57.8)	0.777
Multiparous	1,237 (41.7)	318 (42.4)	563 (41.0)	356 (42.2)	
Delivery mode					
Natural labor	1,885 (63.5)	520 (69.3)	905 (65.9)	460 (54.5)	<0.001
Cesarean section	1,082 (36.5)	230 (30.7)	468 (34.1)	384 (45.5)	
HDP	132 (4.4)	20 (2.7)	52 (3.8)	60 (7.1)	<0.001
GDM	461 (15.5)	199 (26.5)	173 (12.6)	89 (10.5)	<0.001
Infant					
Males	1,531 (51.6)	373 (49.7)	732 (53.3)	426 (50.5)	0.213
Birthweight (g)	3202.5 ± 427.9	3056.5 ± 427.3	3199.6 ± 395.7	3337.0 ± 435.9	<0.001
Birth length (cm)	49.5 ± 1.9	49.0 ± 2.1	49.5 ± 1.7	49.9 ± 1.9	<0.001
Gestational week (weeks)	39.2 ± 1.4	39.0 ± 1.6	39.3 ± 1.3	39.5 ± 1.3	<0.001
Homocysteine	6.19 ± 1.15	6.19 ± 1.12	6.23 ± 1.19	6.13 ± 1.10	0.170

*Data were shown as Mean ± SD or n (%); BMI, body mass index; HDP, hypertensive disorders of pregnancy; GDM, gestational diabetes mellitus; P-values for the differences among GWG categories were estimated using parametric or non-parametric methods, respectively, for continuous or categorical variables*.

### GWG categories in relation to adverse birth outcomes

In our study population, the prevalence rates were 4.9% (*n* = 144), 1.7% (*n* = 49), 14.6% (*n* = 432), and 5.0% (*n* = 149) for LBW, macrosomia, SGA, and LGA, respectively (Table 2). According to the IOM criteria, 41.0% LBW cases (*n* = 59), 30.6% macrosomia cases (*n* = 15), 46.1% SGA cases (*n* = 199), and 30.2% LGA cases (*n* = 45) were included in the sufficient group for GWG, while the insufficient group consisted of 43.1% LBW cases (*n* = 62), no macrosomia cases, 36.3% SGA cases (*n* = 157), and 8.7% LGA cases (*n* = 13). After adjusting for potential confounders, women who had GWG below IOM guideline were more likely to have SGA [adjusted odds ratio (OR) = 1.68, 95% confidence interval (CI): 1.32–2.13] and less likely to have LGA (adjusted OR = 0.51, 95% CI: 0.27–0.96) than those who had GWG within IOM. There were 16.0% LBW cases (*n* = 23), 69.4% macrosomia cases (*n* = 34), 17.6% SGA cases (*n* = 76), and 61.1% LGA cases (*n* = 91) were included in the excessive group for GWG. Compared to those in the sufficient group, pregnant women with GWG above the IOM recommended range had decreased odds of SGA (adjusted OR = 0.60, 95% CI: 0.45–0.79). In addition, higher risks of macrosomia (adjusted OR = 3.47, 95% CI: 1.86–6.48) and LGA (adjusted OR = 3.25, 95% CI: 2.23–4.74) were also observed among pregnant women in the excessive group. Different pre-pregnancy BMI categories, according to WHO standards, were introduced to assess the robustness of our results.

**Table 2:** Associations between adverse birth outcomes and GWG categories according to IOM guidelines.

**Adverse birth outcomes**	**Case/control**			**Adjusted ORs and 95% CIs**	
	**Below**	**Within**	**Above**	**Below vs. within**	**Above vs. within**
LBW^a^	62/566	59/1,123	23/676	1.44 (0.87–2.39)	0.54 (0.29–1.01)
Macrosomia^a^	0/566	15/1,123	34/676	NA	3.47 (1.86–6.48)^*^
SGA^b^	157/566	199/1,123	76/676	1.68 (1.32–2.13)^*^	0.60 (0.45–0.79)^*^
LGA^b^	13/566	45/1,123	91/676	0.51 (0.27–0.96)^*^	3.25 (2.23–4.74)^*^

a *The models were adjusted for education, maternal age, parity, gestational age at delivery, delivery mode, infant sex, HDP, GDM, and homocysteine*.

b *The models were adjusted for education, maternal age, parity, delivery mode, infant sex, HDP, GDM, and homocysteine*.

* *P <0.05; NA, non-available*.

### *MTHFR* polymorphisms in relation to adverse birth outcomes

The minor allele frequency was 23.3% for *MTHFR* A1298C and 28.3% for *MTHFR* C677T in the control group, and all polymorphisms were consistent with HWE (*P* for HWE = 0.266 for A1298C and *P* for HWE = 0.065 for C677T). Dominant, recessive, and additive models were used to assess the association between *MTHFR* polymorphism and the 4 birth outcomes (Supplementary Tables S1, S2). According to the results from crude and adjusted regression models, no significant association was observed between *MTHFR* A1298C and C677T in relation to LBW, macrosomia, SGA and LGA (all *P* > 0.05).

### Modification effects of *MTHFR* polymorphisms on the association of GWG with adverse birth outcomes

Modification effects of *MTHFR* polymorphisms on the association between GWG categories and birth outcomes were explored by dividing the study population according to various genotypes of *MTHFR* A1298C and C677T. As for the A1298C polymorphisms, although no interaction effect was suggested (*P* for interaction > 0.05), obvious differences were suggested between genotypes (see Table 3). Increased risks of LBW (adjusted OR = 2.10, 95% CI: 1.08–4.10) and SGA (adjusted OR = 1.95, 95% CI: 1.43–2.65) were only observed among women with the A1298C AA genotype and GWG below IOM guideline, while no significant association was found among women with the A1298C AC+CC genotype. Similarly, a higher risk of macrosomia was only observed in women with higher GWG and A1298C AA genotype (adjusted OR = 4.19, 95% CI: 1.89–9.29). With regard to the decreased odds of LGA in women with insufficient GWG, no significant association was observed when the population was stratified by the A1298C and C677T genotypes (see Table 4). When the population was stratified by IOM GWG categories, we found pregnant women with insufficient GWG and A1298C AA genotype had an increased risk of 3.96 (95% CI: 1.57–10.01) for LBW, compared with the AA+AC group, while no significant association of C677T polymorphism was observed (Table 5, Supplementary Table S3). Dose-response relationship between GWG values and 4 adverse birth outcomes with spline smoothing function among pregnant women with *MTHFR* A1298C AA genotype was shown in Supplementary Figure S2. A negative correlation of GWG was suggested in relation to LBW and SGA, while a positive correlation of GWG was indicated in relation to macrosomia and LGA.

**Table 3:** Associations of GWG categories and adverse birth outcomes stratified by *MTHFR* A1298C polymorphisms.

**Adverse birth outcomes**	**Case/control**			**Adjust OR (95% CI)**		***P*** **for interaction^c^**
	**Below**	**Within**	**Above**	**Below vs. within**	**Above vs. within**	
LBW^a^						
AA	43/326	34/677	13/401	2.10 (1.08–4.10)^*^	0.55 (0.24–1.25)	0.560
AC+CC	19/240	25/446	10/275	0.80 (0.33–1.96)	0.55 (0.21–1.44)	
Macrosomia^a^						
AA	0/326	9/677	25/401	NA	4.19 (1.89–9.29)^*^	0.331
AC+CC	0/240	6/446	9/275	NA	2.37 (0.80–6.99)	
SGA^b^						
AA	102/326	113/677	42/401	1.95 (1.43–2.65)^*^	0.60 (0.41–0.87)^*^	0.635
AC+CC	55/240	86/446	34/275	1.37 (0.93–2.02)	0.57 (0.37–0.88)^*^	
LGA^b^						
AA	6/326	27/677	60/401	0.41 (0.17–1.03)	3.49 (2.15–5.67)^*^	0.615
AC+CC	7/240	18/446	31/275	0.65 (0.26–1.60)	2.78 (1.51–5.12)^*^	

a *The models were adjusted for education, maternal age, parity, gestational age at delivery, delivery mode, infant sex, HDP, GDM, and homocysteine*.

b *The models were adjusted for education, maternal age, parity, delivery mode, infant sex, HDP, GDM, and homocysteine*.

c *P for interaction was assessed by likelihood ratio test*.

* *P <0.05; NA, non-available*.

**Table 4:** Associations of GWG categories and adverse birth outcomes stratified by *MTHFR* C677T polymorphisms.

**Adverse birth outcomes**	**Case/control**			**Adjust OR (95% CI)**		***P*** **for interaction^c^**
	**Below**	**Within**	**Above**	**Below vs. within**	**Above vs. within**	
LBW^a^						
CC	31/298	36/592	13/350	1.01 (0.49–2.05)	0.60 (0.27–1.33)	0.928
CT+TT	31/269	23/531	10/326	2.26 (0.99–5.00)	0.46 (0.16–1.29)	
Macrosomia^a^						
CC	0/298	9/592	18/350	NA	3.32 (1.44–7.68)^*^	0.600
CT+TT	0/268	6/531	16/326	NA	4.39 (1.63–11.82)^*^	
SGA^b^						
CC	80/298	109/592	41/350	1.54 (1.11–2.15)^*^	0.58 (0.39–0.85)^*^	0.894
CT+TT	77/268	90/531	35/326	1.86 (1.31–2.64)^*^	0.62 (0.41–0.95)^*^	
LGA^b^						
CC	5/298	20/592	46/350	0.44 (0.16–1.20)	3.90 (2.25–6.77)^*^	0.380
CT+TT	8/268	25/531	45/326	0.60 (0.26–1.38)	2.81 (1.67–4.75)^*^	

a *The models were adjusted for education, maternal age, parity, gestational age at delivery, delivery mode, infant sex, HDP, GDM, and homocysteine*.

b *The models were adjusted for education, maternal age, parity, delivery mode, infant sex, HDP, GDM, and homocysteine*.

c *P for interaction was assessed by likelihood ratio test*.

* *P <0.05; NA, non-available*.

**Table 5:** Associations of *MTHFR* A1298C polymorphisms and adverse birth outcomes stratified by IOM GWG categories.

**Adverse birth outcomes**	**A1298C AC+CC genotype**		**A1298C AA genotype**	
	**Case/control**	**OR (95% CI)**	**Case/control**	**OR (95% CI)**
LBW^a^				
Insufficient group	43/326	1.00 (Reference)	19/240	3.96 (1.57–10.01)^*^
Sufficient group	34/677	1.00 (Reference)	25/446	0.93 (0.47–1.83)
Excessive group	13/401	1.00 (Reference)	10/275	0.78 (0.25–2.43)
Macrosomia^a^				
Insufficient group	0/326	1.00 (Reference)	0/240	NA
Sufficient group	9/677	1.00 (Reference)	6/446	1.07 (0.37–3.11)
Excessive group	25/401	1.00 (Reference)	9/275	1.79 (0.79–4.05)
SGA^b^				
Insufficient group	102/326	1.00 (Reference)	55/240	1.35 (0.92–1.97)
Sufficient group	113/677	1.00 (Reference)	86/446	0.90 (0.66–1.23)
Excessive group	42/401	1.00 (Reference)	34/275	0.84 (0.51–1.37)
LGA^b^				
Insufficient group	6/326	1.00 (Reference)	7/240	0.69 (0.22–2.14)
Sufficient group	27/677	1.00 (Reference)	18/446	1.02 (0.55–1.89)
Excessive group	60/401	1.00 (Reference)	31/275	1.30 (0.81–2.08)

a *The models were adjusted for education, maternal age, parity, gestational age at delivery, delivery mode, infant sex, HDP, GDM, and homocysteine*.

b *The models were adjusted for education, maternal age, parity, delivery mode, infant sex, HDP, GDM, and homocysteine*.

* *P < 0.05; NA, non-available*.

### Sensitivity analyses

To assess the robustness of our results, sensitivity analyses were performed by repeating the analyses according to WHO criteria of BMI classifications. The obtained results were similar to the main analyses, and did not drastically change (Supplementary Tables S4–S7, Supplementary Figure S1).

## Discussion

The prevalence of underweight, overweight and obesity for Chinese women before pregnancy in the present study were 19.7, 10.5, and 1.9%, respectively. Excessive GWG accounted for 28.4% of the participants based on the IOM standards, and the proportion was 25.3% for insufficient GWG. Compared to those with normal GWG, pregnant women with insufficient GWG were more likely to give birth to SGA and less likely to give birth to LGA babies, whereas pregnant women with excessive GWG had decreased odds of SGA. Pregnant women in the excessive group had a higher risk of macrosomia and LGA. Interestingly, significant associations of GWG categories in relation to LBW, macrosomia and SGA were only suggested among pregnant women with the *MTHFR* A1298C AA genotype. Among pregnant women with insufficient GWG group, an elevation in the risk of LBW was observed among subjects with the A1298C AA genotype compared to the AC+CC genotype group.

Accumulating evidence has suggested that Chinese people are likely to have higher percentages of body fat (20, 21) and higher rates of hypertension, type 2 diabetes, and dyslipidemia than Caucasian people at specific BMI values (22). The Working Group on obesity in China recommended BMI cutoffs of 24.0 kg/m^2^ to define overweight and 28.0 kg/m^2^ to define obesity (15), supported by evidence from the China Kadoorie Biobank (23, 24). In the present study, 12.4% women were overweight and obesity before pregnancy on the basis of Chinese standards, compared to 8.0% according to WHO criteria. The distributions were comparable with those of previous studies in Chinese women of reproductive age (25, 26), but lower than those in the USA (27) and several European countries (UK, Spain, Belgium, etc.) (28). Our findings did not support the evidence that Chinese BMI standards establish better sensitivity and specificity for identifying adverse birth outcomes than the WHO criteria because the direction and the strength of the obtained associations were similar according to these two standards. This is because of the analogous distributions in the GWG categories. In our study, 25.3 and 28.4% of the women showed insufficient and excessive GWG according to Chinese BMI standards, and the proportions were 26.6 and 26.7% with regard to WHO criteria. The GWG categories in the present study were different from other Chinese studies (15.2% < recommended range and 52.1% > recommended range) (26) and the US population (21.2% < recommended range and 51.0% > recommended range) (27).

GWG is mainly attributed to maternal fat accumulation, fluid expansion, and fetal, placental, and uterine development, which can partly reflect maternal nutrition and fetal development. It is reported that GWG is closely related to a majority of neonatal risks including fetal growth restriction, premature birth, GDM, HDP, and infant mortality, as well as with long-term offspring metabolic health outcomes (29). In the present study, excessive GWG was found to increase the OR for macrosomia and LGA, and decreased the OR for SGA, consistent with the previous studies. For instance, Gou et al. explored the associations of GWG categories with adverse birth weight in a Chinese population of pregnant women with GDM (*n* = 1,523) and demonstrated that excessive GWG could significantly increase a 2.20- and 2.06-fold risk, respectively, for macrosomia and LGA, and decrease the risk of SGA by 51.0% (30). Zhao et al. analyzed the data from 1,617 pregnant women and concluded that excessive GWG was associated with macrosomia and LGA, but no significant association was observed between excessive GWG and SGA risk (26). In addition, our study suggested that pregnant women with inadequate GWG had a higher frequency of SGA (adjusted OR = 1.68, 95% CI: 1.32–2.13) and a lower rate of LGA (adjusted OR = 0.51, 95% CI: 0.27–0.96). Similar conclusions were also reported in studies conducted in China (26), Japan (31), and Norway (32). Our conclusions were supported by a meta-analysis that included more than 1 million pregnancies. Excessive GWG was related to a lower risk of SGA (OR = 0.66), and higher risks of LGA (OR = 1.85) and macrosomia (OR = 1.95), while insufficient GWG was correlated with a higher risk of SGA (OR = 1.53) and a lower risk of LGA (OR = 0.59) (33). Similar to our findings, the relationship between the GWG categories and LBW risk was not statistically significant in this meta-analysis. However, Zhao et al. (26) and Hung et al. (34) reported that insufficient GWG increased the LBW risk. Considering the crucial role of the gestational week on fetal growth, SGA and LGA are thought to be more valuable outcomes compared to LBW and macrosomia, calculated by crude birthweight.

MTHFR is a crucial enzyme that catalyzes the conversion of 5,10-methylenetetrahydrofolate to 5-methyltetrahydrofolate, an important enzymatic process in folate metabolism and the remethylation of homocysteine to methionine. Two common single nucleotide polymorphisms, C677T and A1298C, are known to affect the enzyme function and homocysteine metabolism and have shown potential clinical significance. C677T causes an alanine to valine substitution, resulting in the thermolability of MTHFR. The specific enzyme activity in C677T homozygous mutated subjects decreased to 30% compared to that in heterozygous subjects (~65%) and non-mutated controls (100%) (35). Similarly, the A1298C polymorphism encodes glutamate to alanine substitution, leading to a decrease in enzyme activity to a lesser extent (36). Increased plasma homocysteine levels have been associated with the C677T polymorphism alone and in combination with the A1298C mutation (37, 38). In addition to maintaining a normal range of folate and homocysteine levels, the MTHFR enzyme is important in many biological reactions, including DNA synthesis, cell growth, implantation and invasion of the embryo, especially in fetal growth during pregnancy (39). Recent observations have indicated that *MTHFR* variants are independent risk factors for adverse birth outcomes. For instance, Tiwari et al. in an Indian population found that pregnant women with *MTHFR* C677T mutated subjects have an increased risk for preterm delivery, negative pregnancy outcomes, and LBW (40). Mo et al. conducted a study on two Chinese populations, indicating that the frequency of *MTHFR* A1298C CC genotype was significantly different between a group with adverse birth outcomes and healthy controls (41). However, other studies had reported contrary conclusions (42, 43), consistent with our findings. The present study suggested null associations between *MTHFR* polymorphisms and adverse birth outcomes, neither in crude models nor fully adjusted models. The inconsistent conclusions could be ascribed to the relatively low-frequency distribution of homozygous mutation of *MTHFR* C677T and A1298C and the small sample size in our study. In addition, this might be due to the small difference of homocysteine levels across genotypes. In our study, homocysteine concentrations among *MTHFR* C677T homozygous mutated subjects were comparable with heterozygous subjects (TT: 6.31 ± 1.23 μmol/L vs. CT: 6.32 ± 1.19 μmol/L), slightly higher than non-mutated participants (CC: 6.07 ± 1.10 μmol/L) (*P* < 0.001). With regard to A1298C mutation, consistent with a previous study (37), no significant elevation in homocysteine concentrations was observed, even for homozygous mutant subjects (CC: 6.10 ± 0.95 μmol/L vs. AC: 6.18 ± 1.20 μmol/L vs. AA: 6.20 ± 1.14 μmol/L, *P* = 0.559).

Homocysteine is crucial for the transfer of methyl groups in the activated methyl cycle. Genetic and animal studies had provided clues that homocysteine might regulate the expression of genes involved in body fat storage and lipid metabolisms *via* epigenetic mechanisms (13, 14). Recent evidence from human studies has indicated a potential relationship between *MTHFR* polymorphisms and obesity/overweight. According to the results of a meta-analysis of 38,317 participants, a strong relationship was suggested between homocysteine concentrations and obesity *via* the effect of *MTHFR* C677T polymorphism (TT vs. CC: OR = 1.13, 95% CI = 1.03–1.24) (8). Renzo et al. conducted a dietary study in an Italy population, and observed that participants with C677T CT or TT genotype had higher body weight, BMI, waist, abdomen, hip, waist/hip, fat and lean at baseline, and the ratio of total body lean to total body fat was significantly lower in mutated genotype group after dietary intervention (12). Furthermore, a genetic study by Terruzzi et al. suggested that DNA hypomethylation owing to the lower efficiency of polymorphic MTHFR enzymes could regulate the proliferation and differentiation of myoblasts, promoting muscle growth and increasing muscle mass (44). Considering the alteration effect of *MTHFR* polymorphisms on fat storage and lipid metabolisms, we hypothesized that *MTHFR* polymorphisms possess a potential modification effect on the association between GWG and adverse birth outcomes.

Surprisingly, according to our results, it was A1298C, not the C677T polymorphism, which showed modification effect on the association between GWG categories and adverse birth outcomes, as A1298C does not result in either a thermolabile protein or severely change homocysteine levels in the blood. Significant associations of insufficient GWG in relation to LBW and SGA and excessive GWG in relation to macrosomia were merely observed among pregnant women with A1298C AA genotype. These findings were further supported by the results of the subgroup analysis by the GWG category. Among pregnant women with insufficient GWG, an increased risk of LBW was suggested for A1298C non-mutated women compared to the mutated group, although null associations were indicated for SGA and macrosomia. It could be interpreted that A1298C non-mutated pregnant women were more susceptible to adverse birth outcomes than the mutant participants. Relevant functional studies on the A1298C polymorphism are scarce. The risk effect of the A1298C non-mutated genotype may be ascribed to the influence of the C677T polymorphisms. The *MTHFR* A1298C and C677T polymorphisms show a high degree of linkage disequilibrium (45). According to our data, there were, respectively, 43.2% C677T CT and 15.1% TT genotypes among A1298C AA subjects; and the percentages were 36.9 and 0.6% among A1298C AC subjects and 2.2 and 0.0% among A1298C CC subjects. A1298C non-mutated pregnant women were more likely to be homozygous or heterozygous mutant for the C677T polymorphism. Thus, A1298C non-mutated pregnant women might be at risk of the harmful effect of C677T mutated polymorphism, which has been reported to be related to a variety of adverse birth outcomes. However, in the present study, neither a direct effect nor a modification effect of the C677T polymorphism was indicated according to our analysis. Therefore, the above speculation should be further verified in human studies, along with the functional effect of the A1298C polymorphism. Our findings were similar to those of a previous cohort study (*n* = 2,034) by Said et al., who observed a significant reduction in the risk of severe fetal growth restriction among nulliparous women with the *MTHFR* A1298C homozygous polymorphism (46). In contrast, Chedraui et al. found that A1298C homozygous polymorphism was correlated with higher neck and mid-arm circumference and a higher risk of preeclampsia, which might affect birth outcomes (47). However, it should be noted that our findings may be due to chance regarding the relatively small sample size when stratified by genotypes. Further studies conducted in larger populations are required to confirm our conclusions.

Our work has several strengths. A total of 2,967 pregnant women in a Chinese population were included, and the potential association between GWG categories and adverse birth outcomes were explored by adjusting for major confounders. We assessed whether the Chinese BMI standards establish better sensitivity and specificity for identifying adverse birth outcomes than the WHO criteria in a Chinese population. In addition, we investigated, for the first time, whether *MTHFR* polymorphism modify the effect of maternal GWG categories on adverse birth outcomes. However, this study had several limitations. First, as our research had a retrospective design, we could not judge the causality of the observed associations. Therefore, future prospective studies are required. Second, self-reported maternal pre-pregnancy BMI was applied to calculate GWG, raising possible measurement misclassifications. Third, potential confounders such as diet, physical activity, and other genetic factors were not controlled in our analysis models, which might have affected the reliability of our results. In addition, effects of other SNPs in gene *MTHFR*, especially for those SNPs in 3′ or 5′ near gene, promoter, untranslated regions and exons, had not been studied, which might also influence the study associations. Future studies should focus the modification effects of more variants in gene *MTHFR*. Finally, all subjects were from Han population, and most of them had a high education level (81.3% of college or above). Therefore, the generalizability of our findings to other populations might be limited.

## Conclusion

In summary, our research provides evidence of a potential association between GWG categories, *MTHFR* polymorphisms, and adverse birth outcomes. Women with insufficient GWG had a significantly increased risk of SGA and decreased risk of LGA. Pregnant women with excessive GWG were less likely to give birth to SGA infants and had higher risks of macrosomia and LGA. In addition, a modification effect of the A1298C polymorphism has been suggested. The A1298C non-mutated genotype is considered a risk factor for adverse birth outcome among pregnant women with insufficient GWG. Our findings contribute to a better understanding of the health effects of GWG and *MTHFR* polymorphisms on birth outcomes. However, further prospective studies with larger sample size are required to confirm these findings.

## Data availability statement

The original contributions presented in the study are included in the article/Supplementary material, further inquiries can be directed to the corresponding author.

## Ethics statement

This study protocol was approved by the Medical Ethical Committees of Guangdong Women and Children Hospital. The patients/participants provided their written informed consent to participate in this study.

## Author contributions

XM contributed to the conception and design of the study. WW and DL conducted data analysis and wrote the manuscript. WL, XR, CG, and KL contributed to the analysis and interpretation of the data. All authors have critically reviewed the manuscript for important intellectual content and approved of the final version for publication.

## Funding

This research was funded by the Natural Science Foundation of Guangdong Province of China (2020A1515010434), the Medical Scientific Research Foundation of Guangdong Province of China (A2020059), the Guangzhou Basic and Applied Basic Research Foundation (202102021190), and the National Natural Science Foundation of China (42107450).

## Conflict of interest

The authors declare that the research was conducted in the absence of any commercial or financial relationships that could be construed as a potential conflict of interest.

## Publisher's note

All claims expressed in this article are solely those of the authors and do not necessarily represent those of their affiliated organizations, or those of the publisher, the editors and the reviewers. Any product that may be evaluated in this article, or claim that may be made by its manufacturer, is not guaranteed or endorsed by the publisher.
