# Supplementary material for: Polymorphisms in gene MTHFR modify the association between gestational weight gain and adverse birth outcomes
Source: Front Nutr. 2022 Aug 8;9:919651. doi: 10.3389/fnut.2022.919651 (PMC9393737; doi:10.3389/fnut.2022.919651)
Supplement: Supplementary file 1 [file Data_Sheet_1.PDF]

Supplementary Material

Polymorphisms in Gene MTHFR Modify the Association Between Gestational Weight Gain and Adverse Birth Outcomes

Weixiang Wu[1†], Dan Luo[2†], Xiaolin Ruan[1], Chunming Gu[1], Weiming Lu[1], Kailing Lian[3], Xiaoping Mu[1*]

[1]Department of Clinical Laboratory, Guangdong Women and Children Hospital, Guangzhou, China

[2]Department of Preventive Medicine, School of Public Health, Guangzhou Medical University, Guangzhou, China

[3]Medical Genetics Center, Guangdong Women and Children Hospital, Guangzhou, China

*** Correspondence:** Xiaoping Mu; muxiaoping721@163.com

[†]These authors have contributed equally to this work and share first authorship

Number of pages: 10;

Number of tables: 7;

Number of figures: 2.

TABLE S1. Relationship between A1298C polymorphism and adverse birth outcomes.

Adverse birth outcomes	Genotype	Case/control	OR (95% CI)	*P*-value (FDR-adjusted)
LBW[a]	AA	90/1404	1.00 (Reference)	
	AC	43/818	0.69 (0.42−1.15)	0.152 (0.608)
	CC	11/143	0.96 (0.41−2.29)	0.931 (0.931)
	Dominant model		0.74 (0.46−1.18)	0.201 (0.393)
	Recessive model		1.09 (0.47−2.56)	0.837 (0.837)
	Additive model		0.84 (0.58−1.22)	0.361 (0.409)
Macrosomia[a]	AA	34/1404	1.00 (Reference)	
	AC	14/818	0.70 (0.37−1.33)	0.278 (0.588)
	CC	1/143	0.34 (0.05−2.55)	0.294 (0.588)
	Dominant model		0.65 (0.35−1.22)	0.181 (0.362)
	Recessive model		0.38 (0.05−2.82)	0.345 (0.690)
	Additive model		0.66 (0.38−1.15)	0.143 (0.286)
SGA[b]	AA	257/1404	1.00 (Reference)	
	AC	143/818	0.94 (0.75−1.18)	0.588 (0.784)
	CC	32/143	1.23 (0.81−1.85)	0.335 (0.784)
	Dominant model		0.98 (0.79−1.21)	0.865 (0.865)
	Recessive model		1.25 (0.84−1.88)	0.275 (0.550)
	Additive model		1.03 (0.87−1.22)	0.767 (0.829)
LGA[b]	AA	93/1404	1.00 (Reference)	
	AC	51/818	0.91 (0.63−1.30)	0.603 (0.661)
	CC	5/143	0.59 (0.23−1.50)	0.270 (0.658)
	Dominant model		0.87 (0.61−1.23)	0.421 (0.480)
	Recessive model		0.61 (0.24−1.54)	0.298 (0.375)
	Additive model		0.85 (0.63−1.15)	0.290 (0.351)

[a] The models were adjusted for education, maternal age, parity, gestational age at delivery, delivery mode, infant sex, HDP, GDM, and homocysteine.

[b] The models were adjusted for education, maternal age, parity, delivery mode, infant sex, HDP, GDM, and homocysteine.

TABLE S2. Relationship between C677T polymorphism and adverse birth outcomes.

Adverse birth outcomes	Genotype	Case/control	OR (95% CI)	*P*-value (FDR-adjusted)
LBW	CC	80/1240	1.00 (Reference)	
	CT	55/913	0.83 (0.51−1.34)	0.442 (0.779)
	TT	9/212	0.78 (0.33−1.87)	0.584 (0.779)
	Dominant model		0.82 (0.52−1.30)	0.393 (0.393)
	Recessive model		0.85 (0.36−1.98)	0.701 (0.837)
	Additive model		0.86 (0.60−1.23)	0.409 (0.409)
Macrosomia	CC	27/1240	1.00 (Reference)	
	CT	16/913	0.79 (0.42−1.49)	0.469 (0.625)
	TT	6/212	1.07 (0.42−2.68)	0.891 (0.891)
	Dominant model		0.85 (0.48−1.52)	0.583 (0.583)
	Recessive model		1.17 (0.48−2.85)	0.726 (0.726)
	Additive model		0.95 (0.61−1.46)	0.805 (0.805)
SGA	CC	230/1240	1.00 (Reference)	
	CT	161/913	0.93 (0.74−1.16)	0.503 (0.784)
	TT	41/212	1.04 (0.72−1.50)	0.841 (0.841)
	Dominant model		0.95 (0.77−1.17)	0.613 (0.865)
	Recessive model		1.07 (0.75−1.53)	0.699 (0.699)
	Additive model		0.98 (0.84−1.15)	0.829 (0.829)
LGA	CC	71/1240	1.00 (Reference)	
	CT	60/913	1.09 (0.75−1.56)	0.661 (0.661)
	TT	18/212	1.32 (0.76−2.29)	0.329 (0.658)
	Dominant model		1.13 (0.80−1.59)	0.480 (0.480)
	Recessive model		1.27 (0.75−2.15)	0.375 (0.375)
	Additive model		1.13 (0.88−1.45)	0.351 (0.351)

Note: The models were adjusted for education, maternal age, parity, gestational age at delivery, delivery mode, infant sex, HDP, GDM, pre-pregnancy BMI, and homocysteine.

TABLE S3. Associations of *MTHFR* C677T polymorphisms and adverse birth outcomes stratified by IOM GWG categories.

Adverse birth outcomes	A1298C AC+CC genotype		A1298C AA genotype	
	Case/control	OR (95% CI)	Case/control	OR (95% CI)
LBW[a]				
Insufficient group	31/298	1.00 (Reference)	31/268	1.45 (0.64−3.27)
Sufficient group	36/592	1.00 (Reference)	23/531	0.74 (0.37−1.49)
Excessive group	13/350	1.00 (Reference)	10/326	0.49 (0.15−1.62)
Macrosomia[a]				
Insufficient group	0/298	1.00 (Reference)	0/268	NA
Sufficient group	9/592	1.00 (Reference)	6/531	0.91 (0.44−1.88)
Excessive group	18/350	1.00 (Reference)	16/326	0.66 (0.23−1.92)
SGA[b]				
Insufficient group	80/298	1.00 (Reference)	77/268	0.98 (0.68−1.41)
Sufficient group	109/592	1.00 (Reference)	90/531	0.93 (0.57−1.52)
Excessive group	41/350	1.00 (Reference)	35/326	0.93 (0.68−1.26)
LGA[b]				
Insufficient group	5/298	1.00 (Reference)	8/268	1.82 (0.57−5.80)
Sufficient group	20/592	1.00 (Reference)	25/531	0.98 (0.62−1.53)
Excessive group	46/350	1.00 (Reference)	45/326	1.35 (0.73−2.48)

[a] The models were adjusted for education, maternal age, parity, gestational age at delivery, delivery mode, infant sex, HDP, GDM, and homocysteine.

[b] The models were adjusted for education, maternal age, parity, delivery mode, infant sex, HDP, GDM, and homocysteine.

TABLE S4. Basic characteristics of study population based on WHO BMI standards.

Characteristics	Total (n=2967)	Below (n=750)	Within (n=1373)	Above (n=844)	*P*-value
Mothers					
Maternal age (years)	30.0 ± 3.9	30.4 ± 4.1	29.9 ± 3.8	29.8 ± 4.0	0.005
Pre-pregnancy BMI (kg/m^2)	20.8 ± 2.8	20.7 ± 2.6	20.4 ± 2.6	21.5 ± 3.1	< 0.001
Pre-pregnancy BMI (kg/m^2)					< 0.001
Underweight (< 18.5)	585 (19.7)	180 (22.8)	319 (23.0)	86 (10.9)	
Normal-weight (18.5-24.9)	2146 (72.3)	576 (73.0)	981 (70.7)	589 (74.5)	
Overweight (25.0-29.9)	213 (7.2)	31 (3.9)	82 (5.9)	100 (12.6)	
Obesity (≥ 30.0)	23 (0.8)	2 (0.3)	5 (0.4)	16 (2.0)	
Pregnancy BMI (kg/m^2)	26.3 ± 3.1	24.3 ± 2.3	25.9 ± 2.3	28.9 ± 3.0	< 0.001
Pregnancy BMI (kg/m^2)					< 0.001
Underweight (< 18.5)	2 (0.1)	2 (0.3)	0 (0.0)	0 (0.0)	
Normal-weight (18.5-24.9)	870 (29.3)	434 (55.0)	415 (29.9)	21 (2.7)	
Overweight (25.0-29.9)	1756 (59.2)	345 (43.7)	897 (64.7)	514 (65.0)	
Obesity (≥ 30.0)	339 (11.4)	8 (1.0)	75 (5.4)	256 (32.4)	
Education level					
< high school	149 (5.0)	35 (4.4)	76 (5.5)	38 (4.8)	0.763
high school	405 (13.7)	113 (14.3)	182 (13.1)	110 (13.9)	
≥ college	2413 (81.3)	641 (81.2)	1129 (81.4)	643 (81.3)	
Parity					
Nulliparous	1730 (58.3)	452 (57.3)	814 (58.7)	464 (58.7)	0.777
Multiparous	1237 (41.7)	337 (42.7)	573 (41.3)	327 (41.3)	
Delivery mode					
Natural labor	1885 (63.5)	545 (69.1)	902 (65.0)	438 (55.4)	< 0.001
Cesarean section	1082 (36.5)	244 (30.9)	485 (35.0)	353 (44.6)	
HDP	132 (4.4)	21 (2.7)	53 (3.8)	58 (7.3)	< 0.001
GDM	461 (15.5)	218 (27.6)	167 (12.0)	76 (9.6)	< 0.001
Infant					
Males	1531 (51.6)	732 (53.3)	373 (49.7)	426 (50.5)	0.213
Birthweight (g)	3202.5 ± 427.9	3063.4 ± 424.3	3204.3 ± 400.3	3338.1 ± 434.7	< 0.001
Birth length (cm)	49.5 ± 1.9	49.0 ± 2.1	49.5 ± 1.7	49.9 ± 1.9	< 0.001
Gestational week (weeks)	39.2 ± 1.4	39.0 ± 1.6	39.3 ± 1.3	39.5 ± 1.3	< 0.001
Homocysteine	6.19 ± 1.15	6.22 ± 1.17	6.20 ± 1.16	6.12 ± 1.10	0.121

Note: Data were shown as Mean ± SD or n (%); HDP, hypertensive disorders of pregnancy; GDM, gestational diabetes mellitus; *P*-values for the differences among GWG categories were estimated using parametric or nonparametric methods respectively for continuous or categorical variables.

TABLE S5. Sensitivity analysis of the associations between adverse birth outcomes and GWG categories based on WHO BMI standards.

Adverse birth outcomes	Case/control			Adjusted ORs and 95% CIs	
	Below	Within	Above	Below vs. Within	Above vs. Within
LBW[a]	63/598	60/1133	21/634	1.36 (0.82−2.25)	0.53 (0.28−1.01)
Macrosomia[a]	0/598	18/1133	31/634	NA	**2.92 (1.60−5.33)***
SGA[b]	163/598	198/1133	71/634	**1.68 (1.32−2.12)***	**0.60 (0.45−0.80)***
LGA[b]	14/598	50/1133	85/634	**0.46 (0.25−0.86)***	**3.00 (2.08−4.34)***

[a] The models were adjusted for education, maternal age, parity, gestational age at delivery, delivery mode, infant sex, hypertensive disorders of pregnancy, gestational diabetes mellitus, and homocysteine.

[b] The models were adjusted for education, maternal age, parity, delivery mode, infant sex, hypertensive disorders of pregnancy, gestational diabetes mellitus, and homocysteine.

* $P < 0.05$; NA, non-available.

TABLE S6. Sensitivity analysis of the associations of GWG categories and adverse birth outcomes stratified by *MTHFR* A1298C polymorphisms based on WHO BMI standards.

Adverse birth outcomes	Case/control			Adjust OR (95% CI)		*P* for interaction[c]
	Below	Within	Above	Below vs. Within	Above vs. Within	
LBW[a]						
AA	43/350	35/674	12/380	**1.86 (1.01−3.62)**[*]	0.60 (0.26−1.37)	0.387
AC+CC	20/248	25/459	9/254	0.81 (0.34−1.94)	0.48 (0.17−1.30)	
Macrosomia[a]						
AA	0/350	11/674	23/380	NA	**3.36 (1.59−7.10)**[*]	0.917
AC+CC	0/248	7/459	8/254	NA	2.25 (0.77−6.56)	
SGA[b]						
AA	106/350	111/674	40/380	**1.93 (1.43−2.62)**[*]	**0.61 (0.42−0.90)**[*]	0.508
AC+CC	57/248	87/459	31/254	1.39 (0.95−2.03)	**0.55 (0.35−0.87)**[*]	
LGA[b]						
AA	6/350	30/674	57/380	0.34 (0.14−1.04)	**3.17 (1.98−5.07)**[*]	0.448
AC+CC	8/248	20/459	28/254	0.65 (0.28−1.52)	**2.60 (1.42−4.75)**[*]	

[a] The models were adjusted for education, maternal age, parity, gestational age at delivery, delivery mode, infant sex, hypertensive disorders of pregnancy, gestational diabetes mellitus, and homocysteine.

[b] The models were adjusted for education, maternal age, parity, delivery mode, infant sex, hypertensive disorders of pregnancy, gestational diabetes mellitus, and homocysteine.

[c] *P* for interaction was assessed by likelihood ratio test.

[*] $P < 0.05$; NA, non-available.

TABLE S7. Sensitivity analysis of the associations of GWG categories and adverse birth outcomes stratified by *MTHFR* C677T polymorphisms based on WHO BMI standards.

Adverse birth outcomes	Case/control			Adjust OR (95% CI)		*P* for interaction[c]
	Below	Within	Above	Below vs. Within	Above vs. Within	
LBW[a]						
CC	31/312	37/599	12/329	0.93 (0.46−1.88)	0.52 (0.23−1.19)	0.744
CT+TT	32/286	23/534	9/305	2.17 (0.98−4.73)	0.50 (0.17−1.44)	
Macrosomia[a]						
CC	0/312	11/599	16/329	NA	**2.68 (1.20−5.99)***	0.645
CT+TT	0/286	7/534	15/305	NA	**3.95 (1.52−10.22)***	
SGA[b]						
CC	83/312	109/599	38/329	**1.54 (1.11−2.14)***	**0.57 (0.38−0.85)***	0.905
CT+TT	80/286	89/534	33/305	**1.86 (1.32−2.63)***	**0.64 (0.42−0.98)***	
LGA[b]						
CC	5/312	24/599	42/329	0.35 (0.13−1.01)	**3.26 (1.93−5.53)***	0.799
CT+TT	9/286	26/534	43/305	0.60 (0.27−1.33)	**2.85 (1.69−4.81)***	

[a] The models were adjusted for education, maternal age, parity, gestational age at delivery, delivery mode, infant sex, hypertensive disorders of pregnancy, gestational diabetes mellitus, and homocysteine.

[b] The models were adjusted for education, maternal age, parity, delivery mode, infant sex, hypertensive disorders of pregnancy, gestational diabetes mellitus, and homocysteine.

[c] *P* for interaction was assessed by likelihood ratio test.

[*] $P < 0.05$; NA, non-available.

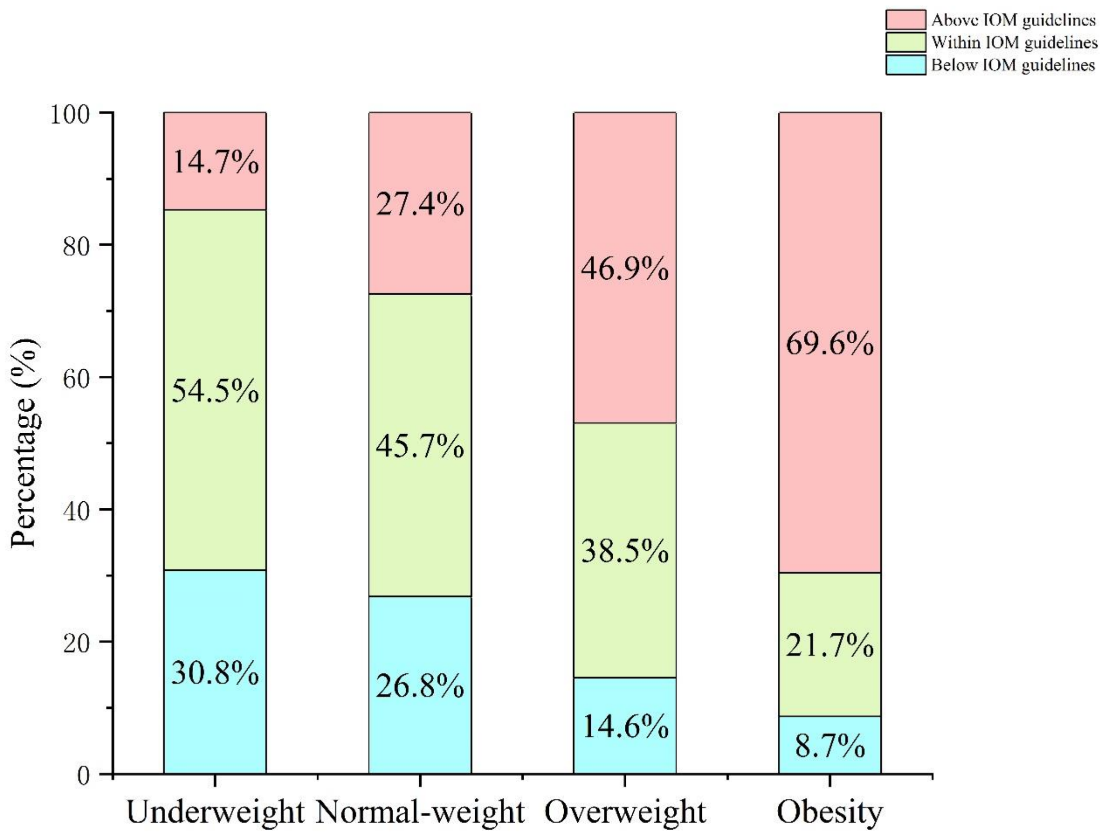

FIGURE S1. Percentage of pregnant women in each WHO BMI category by IOM GWG guidelines. Pre-pregnancy BMI were categorized according to WHO BMI classification (underweight, < 18.5 kg/m^2; normal weight, 18.5 to 24.9 kg/m^2; overweight, 25.0 to 29.9 kg/m^2; and obesity, ≥ 30.0 kg/m^2).

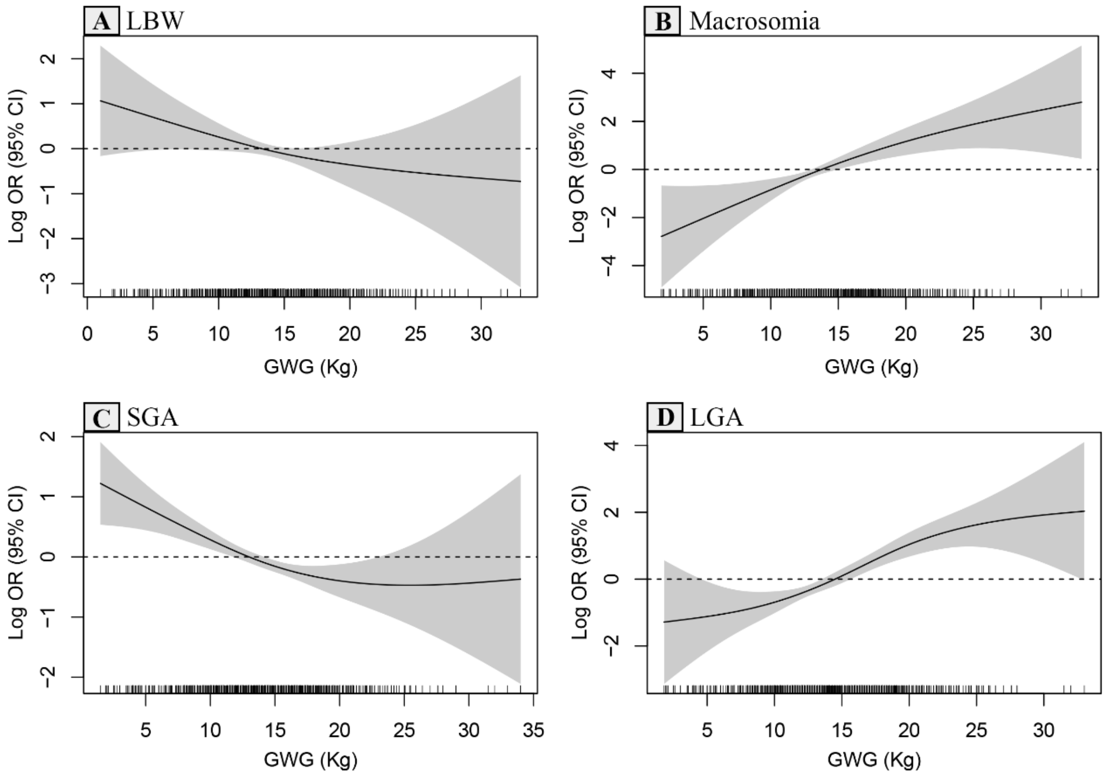

FIGURE S2. Dose-response relationship between GWG values and adverse birth outcomes with spline smoothing function among pregnant women with MTHFR A1298C AA genotype.
